# The Hospital. Nursing Section

**Published:** 1904-11-12

**Authors:** 


### The Hospital.

### Nursing Section.

Contributions for this Section of "The Hospital" should be addressed to the Editor, "The Hospital," Nursing Section, 28 & 29 Southampton Street, Strand, London, W.C.

No. 946.—Vol. XXXVII. *SATURDAY, NOVEMBER* 12, 1904.

### Notes on News from the Nursing World.

### OUR CHRISTMAS DISTRIBUTION.

We have to acknowledge the receipt of a large parcel of articles of clothing for our Christmas Distribution from Miss Taylor, Grove House, College Road, Dulwich, containing two petticoats, a woollen shirt, two pairs of stockings, a night-dress, two shawls, and a blouse. Parcels have also been received from L. R., Hexham-on-Tyne, and S. A. M., Policy 680, Royal National Pension Fund. As one of our kind contributors inquires if she can come to the exhibition of clothing, which takes place a day or two before the parcels are despatched to the hospitals and infirmaries, we may say that she and any other donors are cordially invited to attend. The date of the exhibition will be announced in a future number. We remind our readers that all parcels should be addressed to the Editor, 28 and 29 Southampton Street, Strand, London, W.C., with "Clothing Distribution" written on the outside.

### THE NEED OF MORE MIDWIVES.

At the influential meeting under the auspices of the Association for Promoting the Training and Supply of Midwives, which is reported in another page, Mrs. Humphry Ward called upon every woman of means to support the work of the organisation. The necessity for it could not be more conclusively proved than by the statement of Dr. Cullingworth, who said that though the Midwives Act was passed two years ago the majority of practising midwives in 20 counties had not signified their intention of continuing to practise. This means, of course, that they do not care to work under rules and regulations, but it also means that, unless sufficient trained midwives are available when the five years of grace have expired, the very poor will be no better off than they were before the Act was added to the statute book. It would be a disgrace to the country if the abolition of ignorant midwives were followed by a dearth in the supply of legally qualified women, and the movement upon which the Primate has fitly pronounced his benediction, if adequately supported, will save us from the reproach.

### VICTORIA INSTITUTE, CAPETOWN.

In an interesting sketch of the history of the Victoria Nurses' Institute, at Capetown, which was given by Lady Buchanan at the opening of the new wing by Princess Christian last month, she mentioned that the home was organised as a Diamond Jubilee Memorial to Queen Victoria. The need for the new wing was that in October last year one of the walls of the building fell down, rendering 13 bedrooms useless. With a great commercial depression coming on it was not a good time to appeal to the public; but they had been compelled to do so, with the most satisfactory result that the building was now complete with 26 bedrooms, affording accommodation for 36 beds altogether. The next work they must devote themselves to would be the establishment of a maternity home, which was much needed. Since the opening of the Institute 127 nurses had been entered on the register, and there were 47 on the staff now. During the war 28 went to the front, and six of their nurses were now matrons or assistant matrons in hospitals. One nurse had died of plague while nursing as a volunteer at Maitland, and two had died of typhoid fever. It may be added that all the bedrooms contained in this new wing open on to a balcony and are nice, cheerful rooms, some containing two beds, but the majority having only one each.

### ENGLISH NURSING IN BERLIN.

On Monday a private hospital, which has been specially founded in the interests of English and American patients who desire to receive more individual nursing than is obtainable in the Berlin hospitals, was opened in the German capital. It is under the management of two English hospital nurses, one of whom was trained at St. Bartholomew's Hospital; and the British chaplain, in performing the opening ceremony, naturally spoke warmly in praise of English nursing.

### UNNECESSARY PRESENTATIONS.

A correspondent protests against the increasing tendency of making presentations, and characterises it as "an odious custom." We do not think that this description is justifiable. A presentation to a popular official, who, after many years of admirable and kindly service is resigning an appointment, is a pleasant mode of showing esteem. But we admit that it is possible to make unnecessary presentations, such as some of those instanced by our correspondent, whose experience certainly goes far to warrant irritation. It is not right that nurses should be frequently called upon to subscribe to presentations, and under no circumstances ought they to run the risk of being snubbed or reproached because they are either unwilling or unable to contribute.

### INTERESTING CEREMONY AT DUBLIN.

Last week a ceremony of interest to all past and present members of the nursing staff of Sir Patrick Dun's Hospital, Dublin, took place at the Nurses' Home in Lower Mount Street. The "Margaret Huxley" Prize was awarded for the first time, and presented by Miss Huxley to Miss Kathleen Haire, who has worked at the hospital for five years and is now leaving for an appointment in Belfast. This prize was instituted in 1902 and endowed with a sum of money, subscribed by former Dun's nurses as a mark of their esteem of Miss Huxley's work during a period of 18 years as lady superintendent of the hospital. The prize is to be awarded every two years; it takes the form of a brooch, the coat of arms of the hospital in enamel and gold, with the motto "Celer atque fidelis," and "Margaret Huxley Prize" beneath. On the back is engraved the name of the winner, and "For excellence." The names of the winners are also preserved on a parchment roll of honour, which hangs in the nurses' sitting-room for all to see. In the course of her address to Nurse Haire, Miss Huxley also spoke to those who can look forward to winning the prize in the future; she asked them to take a high ideal and try to live up to it, that by their quiet, dignified bearing they may inspire confidence and respect for their calling. A very pleasant evening was spent; the probationers had arranged a programme of music and dancing, and it was a great pleasure to all to welcome Miss Huxley and the many past nurses who had been able to accept the invitation of the present staff. Among the visitors were Miss MacDonnell, Royal Red Cross lady superintendent of the Richmond Hospital; Miss Hosford, lady superintendent of the Royal Victoria Eye and Ear Hospital; Miss Hughes, lady superintendent of Portrane Asylum, and several others who hold responsible positions in Dublin and the neighbourhood.

### THE MATRON OF HORWICH ISOLATION HOSPITAL.

We understand that there were no fewer than 136 applications for the post of matron of the new hospital at Fall Birch, Horwich. The salary offered was not remarkable, being £65 per year to a person who had not previously been matron, or £70 to any one who had acted in that capacity; and it was essential that applicants should have had at least seven years' experience in nursing. Miss Hughes, who has been appointed, was seven years at Tonbridge Infirmary, and besides her general training in that institution she received fever training at Brighton. She has also been superintendent nurse in a workhouse, matron of an isolation hospital, and assistant matron at Parkhurst, Newport, Isle of Wight. She is, therefore, thoroughly well qualified for the post she has obtained against 135 competitors. The very large number shows that the matronship of a modern infectious diseases hospital has great attractions to qualified nurses.

### THE STRANGERS' HOSPITAL AT RIO.

It will be recollected that in June last we mentioned the disappearance in the previous month of Sister Isobel Grewer from the Strangers' Hospital, Rio de Janeiro, and that a few weeks later we received a letter from the matron stating that the body had never been found. The *Brazilian Review* for October 18, referring to the report of the hospital, lays stress upon the fact that since the loss of Miss Grewer no new nurse had been engaged, so that the staff now consists only of the matron and two sisters. As our contemporary points out, the staff thus reduced is so small that even a slight outbreak of yellow fever might be more than they could cope with. Since the report shows that for the first time in its history the Strangers' Hospital has not only paid its way, but has a balance to the good, it can scarcely be pleaded that the delay in appointing a new sister is due to pecuniary difficulties.

### THE VISITING NURSE IN TUBERCULOUS CASES.

It is an interesting fact that in the first three months of visiting nursing a nurse in the employ of the tuberculous dispensary of the Johns Hopkins Hospital, Baltimore, who attended in that period 65 cases of consumption, induced 38 of the patients to follow with tolerable faithfulness a correct *régime* of life. The figures are the more noteworthy because many of the patients were utterly hopeless cases, almost too ill to appreciate the value of any measures. The experience of this nurse will tend to encourage the movement in America, for the augmentation of the number of out-door clinics exclusively for tuberculous patients, each with one or two visiting nurses. At present Baltimore, Boston, Minneapolis, and Philadelphia have two each, and New York at least five; and the admirable work is rapidly developing.

### A MEMORIAL HOME IN GLASGOW.

The memorial stone has just been laid at Glasgow of the "Alice Mary Corbett" Nurses' Home. The building is in connection with the Samaritan Hospital and the ceremony was appropriately performed by Master Godfrey Corbett, son of the deceased lady to whose memory the home is being erected and grandson of Mrs. Polson, the donor of the building and site. Mrs. Polson has expended no less than £10,000 upon the home. Mr. A. Cameron Corbett, M.P., in acknowledging the vote of thanks which was moved to Mrs. Polson and his son, said that there might be nurses who regarded their calling only as a means of making an income, but if there were he had never met them. They were inspired by something more than that. It was not always where men dared to die that the highest examples of human heroism were to be found, but often in the spheres of daily drudgery. The hospitals were doing much for the bodies of those within their walls, but the kindly touch of the nurses did as much for human life as was done by the doctors in the direction of alleviating pain.

### APPOINTMENTS BY POOR-LAW GUARDIANS.

At the last meeting of the Brighton Guardians a special report from the Workhouse Stock and Management Committee was adopted, with the result that the guardians agreed to apply to the Local Government Board for the issue of an order whereby, in future, it shall not be necessary for them to report to that Board each separate appointment of a nurse at the workhouse, nor to obtain their approval to the remuneration of any such person so appointed. This order they desire to be made applicable to a maximum of 42, so that if at any time they desire to add to the number of the nursing staff, they can appoint two more charge-nurses, two more staff nurses, and two more probationers. Under the order dated February 28, 1871, it is required that each individual appointment, with the salary given, shall be reported to the Local Government Board for approval, and if the application of the Brighton Guardians is complied with similar applications will certainly be addressed to the authorities at Whitehall. We do not think that the latter should lightly part with the power which is vested in them.

### LONDON BIBLEWOMEN AND NURSES' MISSION.

At a meeting held last week in support of the London Biblewomen and Nurses' Mission, founded by Mrs. Ranyard in 1857, Sir Thomas Barlow, who presided, observed that the improvement in nursing in the Victorian era was largely due to the efforts of this and other societies. The nurses employed by the Society were all hospital trained, and worked under the direction of the doctors of the district, while they were in close touch with out-patients' departments of hospitals. He had been most favourably impressed with the type of women employed and the useful character of the work which was carried out by the agents of the Society. Sir William Broadbent also expressed the interest which he felt in the Mission, and said that by the combination of the influences of the Biblewomen and nurses it achieved the maximum amount of good and useful work.

### IRISH NURSES AND PHYSICAL CULTURE.

The first of a series of lectures to the members of the Irish Nurses' Association was given last week by Dr. McVittie on "Physical Culture." Mrs. Kildare Tracey presided, and welcomed him in a few appreciative words. The lecturer, who made use of lantern slides for purposes of illustration, said that he was pleased to have the opportunity of addressing so large a body of nurses, who, above all people, had it in their power to enlighten parents upon the important question of physical culture, and of pointing out the evils resulting from the present-day system of excessive mental pressure upon very young children.

### PROGRESS AT BARNSTAPLE.

At the annual meeting of Barnstaple and District Nursing Association, the welcome announcement was made that the income exceeded the expenditure to the extent of upwards of £23. The President, it is true, had to warn the members that there were several contributions to the fund which must be regarded as exceptional. But there is, at any rate, no reason why at the end of another year a surplus should be converted into a deficiency. The number of cases attended was 175, and the number of visits paid 3,152, a record which proves that the single nurse employed by the Association must have had her hands very full. There was one unsatisfactory feature mentioned at the meeting. Neither the Barnstaple Guardians, nor the Friendly Societies, support the organisation. In many other places there is no ground for this complaint, and the Friendly Societies as a rule are prompt to recognise the value of the work of a trained nurse.

### THE GUILD OF ST. BARNABAS.

A notable service was held last month in connection with the General Convocations of the Church in America, when the National Council of the Guild of St. Barnabas assembled at the Church of the Advent in Boston. The Bishop of the diocese in welcoming the nurses, made a happy allusion to the fact that within half a mile of the Church of the Advent, the first successful use of anæsthetics was made about 60 years ago; and, he added, that as modern surgery is the outcome of the use of anæsthetics, and modern nursing is the outcome of modern surgery, it was peculiarly fitting that the members of the Guild of St. Barnabas should hold their first service in that particular church on the occasion of the meeting of the National Council in Boston. Bishop Brent, who preached the sermon, his subject being "Life, not Death, the Dominating Force," urged that faith in the power of life should go hand in hand with technical skill in nursing.

### FATAL ACCIDENT AT HASTINGS.

An inquest was held last Friday afternoon on the body of Mary Ann Petit Chetwynd, who was found dead the previous morning on a ledge beneath the Fairlight Cliff at Hastings. The most important witness was a magistrate residing at Dolgelly, North Wales, who stated that Miss Chetwynd, who was a great-niece of his mother, was a trained nurse and had been nursing his mother at Rocklands, East Hill. Miss Chetwynd was 33 years old, and the witness stated had been low-spirited since the death of his mother, which occurred on October 7; but neither in his evidence, which shows that she went for a walk with him the afternoon before her body was found, and on parting said she should be in shortly afterwards, nor in that of any other witness, was there anything to justify the theory of suicide. The coastguard who found the body said that it had apparently fallen over the cliff, and lodged on the ledge, which was 150 feet below the top of the cliff. The coroner having expressed his conviction that death was due to accidental circumstances, the jury returned a verdict to that effect.

### THE IMPORTANCE OF A NAME.

The Hampstead Guardians have resolved that, subject to the consent of the Local Government Board, the sick-wards of the workhouse shall in future be styled the Hampstead Workhouse Infirmary, "except in the official returns made to the Local Government Board." The chief reason assigned for the proposed alteration is understood to be that the Guardians consider that the certificates granted for nurses who receive their training at the institution will be of greater value than at present if headed "Hampstead Workhouse Infirmary." The nursing in the sick wards of a workhouse is, or should be, the same as in a separate building; and we do not think that the Local Gevernment Board can do any harm by acceding to the proposal of the Hampstead Guardians. But we doubt whether a change of name makes any difference to the value of a certificate.

### THE SECRETARY OF THE PENSION FUND.

We think that all members of the Royal National Pension Fund for Nurses who do not already possess a photograph of their popular secretary, Mr. Louis Dick, will be glad to learn that the Scientific Press, 28 Southampton Street, Strand, have produced an excellent photograph portrait of him in post-card form. The photograph is a reproduction from the portrait published in the Souvenir Album of the Pension Fund, and is supplied to nurses on the same terms as photographs of the Reception Ceremony at Buckingham Palace.

### The Nursing Outlook.

"From magnanimity, all fear above;
From nobler recompense, above applause,
Which owes to man's short outlook all its charm."

### NURSES AND THE MILK SUPPLY.

It is more evident every day that the high death-rate and high disease-rate of the infants of our big cities must be met by special precautions and special instructions. In what follows it must be remembered, as we stated in our issue of the 29th ult., that each municipality should remove the necessity for sterilising the milk by a system of inspection which will render innocuous every source of milk supply for their locality; a necessity which was very well demonstrated by the evidence given recently in an action for damages brought against a leading dairy company and the chief details of which we publish on another page.

There are many nurses engaged in lecturing on the rearing of infants, in inspecting baby-farms, in acting as "health visitors," and calling on young mothers and giving them good advice. This is all particularly useful where the children are fed by the breast, as they ought to be, but unfortunately it falls to the lot of too many babies to be brought up by hand. And then the proper mixing of the milk, the quantity of each meal, the cleansing of the bottles, and so on, becomes a labour demanding care and attention difficult to procure. Even if all this is taught, and all carried out, there is the source of the milk supply to be taken into consideration; if the ha'p'orth of milk from round the corner is not quite sweet, or has been standing in unclean vessels, or comes from some polluted farmyard, the proper mixing and preparing are not enough. And it is impossible for the mothers in poor districts to sterilise the milk. So it has come about that there is a wide movement in favour of providing milk depôts where properly sterilised and prepared milk in so many bottles per day, according to the age of the infant, can be supplied. The mother has nothing to do but to warm the bottle by standing it in hot water, to remove the stopper and slip on the teat supplied, and then give it to the child. Woolwich, Lambeth, and Finsbury are all going to establish such depôts by municipal means, and Dr. Newman, of Finsbury, is already buying his plant. Newcastle is going to have a private depôt run from philanthropy. Then one hospital and one dispensary in London are thinking of starting similar milk supplies. Now in New York, Rochester, and elsewhere in the United States where this movement is also to the fore, it is usual to place a trained nurse in charge of the depôt, and we are convinced that this should be done in England also.

Consider that what we know as "surgical cleanliness" is absolutely necessary, and that there must be complete accuracy about all measurements and mixings. Then it is part of the rule of the York milk depôt that every mother should be visited and advised about her baby when the milk is first used, and that the child should be visited and its progress reported at subsequent intervals. At the Battersea depôt an attempt is made to have the infants brought to be weighed every week, but it is found somewhat difficult to get the mothers to do this. And, after all, it is very important to secure statistics with regard to the results of the constant use of sterilised milk. It is just possible to forego the risk of infection at too great a cost, and though so far, the statistics of the health of infants reared on sterilised milk are good, it is always well to have a little suspicion with regard to any new enthusiasm. Now, this weighing and watching of the infants is essentially a nurse's work. A third part of the duties of the manager of a milk depôt would be to keep the books and accounts.

Most obviously, a nurse straight from hospital is not fit to be put in charge of a milk depôt, and this is so evident to the managers of the York depôt that they are ready to allow a trained nurse selected by the Editor of this paper to have 10 weeks' free training, so far as they can supply it to her. She would help in the early morning in the preparation of the milk, she would visit the homes, she would be allowed to see the model farm from which the milk comes, she would be supplied with the literature on the subject which it is difficult at present to obtain, and she would be shown how to keep the books. We also feel sure that she would be welcome to see over the Battersea depôt, and comparisons are always valuable. Thereafter it would remain with herself to find a post as manager in one of the newly-formed depôts, and to secure a sufficient salary. So far, the salaries paid have been very good.

Any nurse who feels interested in the attempt to stop physical degeneration by organising a pure milk supply, who has had general training and special training as regards infants, and is anxious to learn the work of a milk depôt, should write to us and send two testimonials and two present references; marking the envelope outside with the words "milk supply." It is work which needs physical health, which needs a sound education, and which needs great tact in dealing with the mothers. It is more fitted to a woman of 30 than to a very young or very old nurse; it needs a steady worker, yet one with enough originality to throw herself into the starting and developing of a new business. It is not exciting like the surgical wards of a hospital, it will be a somewhat lonely and difficult post. There is also so much uncertainty about it, that it is not advisable to approach it merely from the money-making point of view. But we sincerely hope that these posts will in future fall to English nurses here, as they have fallen to American nurses on the other side of the water.

### Lectures upon the Nursing of Infectious Diseases.

By F. J. WOOLLACOTT, M.A., M.D., B.S.Oxon, D.P.H., Senior Assistant Medical Officer, Park Hospital, Metropolitan Asylums Board, Hither Green.

### LECTURE IX.—THE ANTITOXIN TREATMENT OF DIPHTHERIA.

UNTIL about ten years ago the treatment of diphtheria was unsatisfactory and often of little practical use. So fatal was the disease that nearly one-third of the patients admitted to the London fever hospitals died, and there were no methods known by which its progress could be checked. The introduction of the antitoxin treatment in 1894 has effected a great change, and has provided us with a remedy of the highest value which, in spite of much opposition, is now almost universally adopted. Before considering how it is applied and how it acts, it will be of service to explain briefly what is meant by antitoxin and in what its value consists.

Diphtheria, like other infectious diseases, is caused by the action of special microbes shaped like small rods and known as diphtheria bacilli. By some means or other they obtain access to the throat where, if the conditions are favourable, they rapidly multiply and give rise to the membranous exudation and the other local changes previously described. In addition to this the bacilli manufacture a poisonous substance which is absorbed into the blood, and so carried to distant parts. In this way the whole of the body may be affected. The poison has a special tendency to attack the heart muscle and the nervous system, facts which explain the frequency of heart failure and of paralysis. Before recovery can take place the poison, or toxin as it is called, must be destroyed, and fortunately, in many cases, the tissues of the body are able to manufacture an antidote. This antidote is known as antitoxin, and after all the toxin is neutralised a certain quantity of it remains in the blood.

With a knowledge of these facts it seemed likely that as the blood of an animal which has recently passed through the disease contains the antidote, it might be productive of benefit to others still suffering. This was the origin of the antitoxin treatment. The method of preparation is as follows:—A horse is injected at intervals with doses of diphtheria toxin of increasing strength. In the course of time its blood becomes rich in antitoxin. A quantity is then drawn from one of the veins, and allowed to clot. To the clear serum an antiseptic is added, and the result is the fluid which is used in the treatment of diphtheria. In other words, antitoxic serum is the blood serum of a horse which has passed through an artificial attack of diphtheria and made a good recovery. Its value is due to the fact that it contains a substance which is an antidote to the poison of the disease.

Antitoxic serum is administered by subcutaneous injection, as a rule into the abdominal wall or the loins. A special form of syringe is used which can be taken to pieces and easily cleaned. Between its nozzle and the needle it is advisable to insert a piece of rubber-tubing, which will give to any sudden movement of the patient and so prevent the needle being driven into the deeper tissues. Before use the whole apparatus must be sterilised by boiling. The skin should be washed with soap and water, and then with 1 in 20 carbolic lotion. After the injection the skin puncture is sealed with collodion. The little operation is rather painful, especially while the fluid is running in, and a feeling of soreness often persists for some time. After use cold water should be drawn several times through the needle, tube, and syringe till all traces of the serum are removed. They may then be boiled. If put directly into hot water the serum will coagulate and block the apparatus.

Wide differences of opinion still exist as to the quantity that should be given, but on the whole there appears to be a tendency to give it freely, the amount being graduated according to the severity of the disease. Unlike ordinary remedies the age of the patient does not influence the dosage, and children need as much as adults. The strength of the serum is estimated in units, of which 12,000 should be given in the more serious, and 6,000, or rather less, in the milder cases. If these doses be adopted a repetition of the injection is hardly ever necessary.

As has been stated, the introduction of antitoxin marks a very decided improvement in the treatment of diphtheria. Of the resulting benefits the chief is a great reduction in the mortality among the patients, which is less than half of what it formerly was. It is important to remember that the full value of the remedy is not obtained unless it be given early in the disease. If given on the first day the symptoms rapidly clear up, and complete recovery soon follows. Under these circumstances the attack does not become serious. On the other hand, if the disease be allowed to proceed unchecked for any length of time, a large amount of the poison may be absorbed, and irremediable danger result, for the antitoxin, though able to destroy the poison itself, is powerless to undo its harmful effects. Like every other antidote, to be effectual it must be administered at the earliest possible moment. Unfortunately, as we have seen, the onset of diphtheria is often insidious, so that it may be unrecognised at first. If an early diagnosis could be made in every case, the use of the remedy would rarely fail to cure.

The action of the antitoxic serum is twofold, first on the membrane, and secondly on the poisons produced by the bacilli. The effect on the membrane, though not immediate, usually begins after a few hours. It ceases to spread, and begins to loosen, finally disintegrating and dissolving away, or occasionally separating in large pieces. One of the chief dangers of diphtheria in former times was the extension of the disease to the larynx and lungs, causing death from croup or pneumonia. The use of antitoxin has very much diminished this danger, for after its injection the advance of the membrane is soon checked. Even if it be not given till the symptoms of croup have already appeared, great benefit usually results, and recovery is much more frequent now than formerly.

The paralysis, which so often complicates an attack of diphtheria, is comparatively rare when antitoxin is given quite early, but, the longer the treatment is delayed, the more likely is it to manifest itself in some form or other and the more serious is it likely to prove.

In addition to its curative action, antitoxin is occasionally used as a preventative, and after exposure to infection its administration has been shown to be of considerable value in warding off an attack.

Such are the chief benefits resulting from the treatment. Certain disadvantages remain to be considered. It occasionally happens that an abscess develops at the site of injection. In most cases this is due to neglect of antiseptic precautions, and may be avoided by thorough cleansing of the skin and sterilisation of the syringe. Should an abscess appear it must be opened as soon as possible, since, owing to the thickness of the skin, the pus is prevented from coming to the surface and may burrow extensively in the abdominal wall.

Other troublesome and less preventable consequences are various skin eruptions, some of which may be observed in nearly half the cases treated. Besides transient inflammatory changes in the neighbourhood of the injection two forms of rash are common. The first is of the urticarial or nettle-rash type, and, as a rule, begins about the end of a week. It lasts two or three days, coming out in crops, and, apart from the troublesome itching, is of no importance. The other, though less frequent, is more severe. Its onset is later, usually about the end of a fortnight. In character it somewhat resembles measles, consisting of red blotches or rings, which are commonly thickest on the buttocks, knees, and elbows, though the rest of the body does not escape. This rash is not infrequently accompanied by troublesome pain in and round the joints. The latter are slightly swollen and stiff, and the pain is increased on movement. The temperature, too, is usually raised, and albuminuria may make its appearance, or, if already present, may increase in amount. The duration of the symptoms is about three or four days, and the patient often experiences a good deal of discomfort. Relief may be obtained by wrapping the joints in cotton-wool, but at times sedatives are necessary. In patients who have on some previous occasion undergone the antitoxin treatment for another attack of diphtheria the injection may be very closely followed by the rash, sometimes within a few hours, or even a few minutes. It is then usually of an urticarial character, but may closely resemble scarlet fever. There is often a rise of temperature, with perhaps a shivering attack or a rigor, and, in some rare cases—in children—convulsions have ensued. These drawbacks to the use of antitoxin, though by no means slight, are insignificant when compared with the invaluable services that it is capable of rendering. A remedy which saves lives is not to be dispensed with because occasionally it entails some unpleasant consequences.

### The Training of Midwives.

### IMPORTANT MEETING.

On Friday last an important meeting was held at the Westminster Palace Hotel in order to enlist public support for the Association for Promoting the Training and Supply of Midwives. Princess Henry of Battenberg attended the meeting. The chair was taken by the Archbishop of Canterbury; and the company present included Lord Balfour of Burleigh, Lady Montagu, Lady Lucy Hicks-Beach, Lady Flower, Lady Eardley, Lady Sendall, Mrs. Randall Davidson, Mrs. Humphry Ward, Mrs. Scharlieb, M.D., Mrs. Wallace Bruce, Mrs. Raffles Hughes, Mrs. Charles Schwann, Mrs. Schacht, Miss Lucy Robinson, Sir T. Smith, Dr. F. H. Champneys, Dr. Robert Boxall, Dr. C. J. Cullingworth, Mr. and Mrs. Wynne Baxter, the Rev. C. Singer, and Mr. A. L. Leon. Many nurses were also among the audience.

The Archbishop of Canterbury, in his opening address, said that he, personally, could lay no claim to an intimate knowledge of the movement. He wished to welcome the delegates, who had come from many parts of England—from 15 county councils, 12 borough and town councils, and 12 district councils. Most of the representatives were medical officers. Some 60 years since, the Archbishop continued, it was technically required that any woman acting as a midwife in a parish should be licensed by a bishop. He had in his possession such licenses, some 100 years old. This historical peculiarity showed that long ago it was felt that those who did this work should, besides being technically qualified, be also persons of good conduct and character. The meeting that afternoon was held in order to emphasise the responsibility of the community in consequence of the legislation which was passed two years ago. The great need was for an adequate supply of women who were not only qualified midwives, but whose influence was also for good. There was every sign that under the guidance of the Association the work would be properly carried out.

In the absence of Lord Brassey, who sent a telegram regretting his inability to be present, Dr. C. J. Cullingworth moved the following resolution:—

That in view of the urgent need that exists in this country for a supply of properly trained midwives who, in working in conformity with the provisions of the Midwives Act of 1902, will help to reduce the present high mortality of lying-in mothers and their children amongst the poor, this meeting cordially supports the Association for Promoting the Training and Supply of Midwives, and earnestly commends its work to the generous sympathy of all who are interested in the welfare of the country.

Dr. Cullingworth went on to say that since the passing of the Midwives Act of 1902, in 20 counties the majority of practising midwives, not caring to work under rules and regulations, had not signified their intention of continuing to practise. There was, therefore, urgent need to fill the gap and find suitable women trained for the work. The Act gave a breathing-time of five years before any final consequences would fall on women who were practising without proper qualifications; and the Association would endeavour to fill the gap, taking at the same time a larger view of its work, recognising that none but trained and tested women would be allowed to practise, and that careful selection must be made, and women must, if necessary, be assisted in their training.

The Rev. C. Singer, representing the Chief Rabbi—who was not well enough to be present—seconded the resolution.

Dr. F. H. Champneys, chairman of the Central Midwives Board, spoke in support of the resolution. He mentioned that at least four members of the board were in entire sympathy with the objects of the Association. He thought that the Midwives Act was not generally known. Formerly, women had to choose between well-trained, clean nurses, and dirty, ignorant nurses. Now they would not be allowed to choose.

Mr. Wynne Baxter, coroner for the Eastern District of London, mentioned that of 464 inquests he had held upon children under 12 months old, 87 were deaths from smothering in bed, and he had no doubt that the majority of deaths were the result of the ignorance of mothers and midwives.

Miss Lucy Robinson, vice-chairman of the committee, dwelt upon the great dangers and troubles that attend childbirth in the homes of the very poor who are without the skilful aid of doctor or trained nurse.

Dr. W. Williams, medical officer of health for the Glamorgan County Council, also supported the resolution.

Mrs. Humphry Ward signified her earnest sympathy with the movement. She appealed to the wealthier classes to respond to the appeal for subscriptions to the Association. For those who had means childbirth was year by year becoming safer, and childhood and motherhood were surrounded by every care and tenderness provided by love and money. Every woman needed skilled attention at such a time. There were hospitals where skill and kindness were available for the poor, but there were many homes where the lives of the mothers and the lives of the children were at the mercy of ignorance. Those who worked among the poor knew how often a mother, in ignorance, sacrificed herself by neglecting to protect herself, and trusting to unskilled help. Every woman of means should help her poorer sisters by supporting the work of the Association.

Mr. A. T. Leon, hon. treasurer of the Association, having spoken, the resolution was carried unanimously.

The Archbishop of Canterbury having proposed a vote of thanks to Princess Henry of Battenberg for her presence, Lord Balfour of Burleigh proposed a vote of thanks to the Chairman, and these votes were both carried with acclamation.

### Bertie's Drive over Waterloo Bridge and what He Saw There.

A VISIT TO A CHILDREN'S HOSPITAL. BY MRS. MOLESWORTH.

"His childish eyes grew round with pity as he gazed."

It was Christmas holidays—Christmas of 1903. Everybody knows how schoolboys look forward to Christmas holidays, and in this Bertie had certainly not been behindhand. And as he had only been at school for a year, these were his first. But somehow, kind as all his home people were, and in spite of his nice presents, it did not seem quite like Christmas—not so "jolly" as he had expected. I think it was greatly owing to the weather. For weeks it had done nothing but rain, and the days were always sunless and gloomy. There was no hope of skating, and indeed very little temptation to go out at all, unless covered up in a mackintosh and burdened with an umbrella. And Bertie missed his schoolfellows, for whatever the weather is, a lot of boys together are never at a loss for fun.

He had no brother. His sister Maggie was nearly two years younger, and considering she was "only a girl," she was a companion not to be despised in many ways. Then there was baby, a fat, cheery little person of three. And Bertie loved them both dearly, and he loved his home, but still—it was rather dull. He felt glad that he was at school in the country; he was beginning to think he did not like London, any way not in winter. So he stood at the window, an hour or so after breakfast, wondering what he should do with himself in this particularly gloomy and rainy morning. Only another week and he would be back at school; on the whole he didn't much mind. He drummed on the panes and hoped it wouldn't be like this at Easter. Then the door opened and his father looked in, calling to the footman in the hall to whistle for a Hansom at once.

"Why, Bertie," he said, "you look rather down in the mouth, my boy. What's the matter?"

"Nothing—at least—yes, it is nothing—it is that I have nothing to do," and he laughed a little.

Then a sudden idea struck him.

"Oh, dad," he exclaimed, "Do let me go with you. I love Hansoms and if you drop me anywhere in the City, I can find my way home again in the Underground or by 'bus."

His father hesitated.

"I am not going to the City this morning," he replied. "I have an appointment over by Waterloo Bridge—nothing that would amuse you."

But Bertie grew all the more eager.

"I've never been over Waterloo Rridge," he said, "it would amuse me. I'd like it awfully."

"Come along then," was the reply, and by the time Bertie had got his top-coat and hat a Hansom was driving up at the front door. Bertie jumped in, while his father gave his directions. The driver seemed a little at a loss, and made some further inquiry.

"Just straight along the Bridge Road. You can't go wrong. 'Poverty Corner,' they call it."

The man's voice cheered up.

"Poverty Corner," he repeated, "to be sure," and off they started.

"What a queer name!" said Bertie. "Is that where you have an appointment dad?"

His father smiled.

"Yes," he replied, "that's the nickname for the place I am going to. Rather a sad one, don't you think?"

"Yes," said Bertie slowly, for he was not a thoughtless boy. "I think it is. Are the people dreadfully poor about there?"

"Very, very poor," his father answered, "though there is a certain amount of keeping up a respectable appearance which makes it almost sadder."

"And are you going to see some of the poor people there?" asked the boy.

His father shook his head.

"No, I am going to the Children's Hospital there. I have some business with the officials."

Bertie's eyes brightened.

"Oh, I'd like to see it," he said. "I have been at a children's convalescent home; that's the same thing, isn't it? They took some of us over to see it one half holiday at school. It was awfully jolly—such big dormitories and splendid gardens. And most of the children seemed quite happy."

"They were getting well, you see," said his father. "A Home of that kind is a more cheerful sight than an actual hospital."

Still Bertie's imagination scarcely took in the difference. It was full of the beautiful house in the country which he had seen, and after they had crossed the bridge and the river was no longer in view with its interesting sights, he could hardly believe they had reached their destination when the cab drew up in front of a small house—one of a commonplace row—just round a corner from the main road.

"Why, I thought—" he began—but his father seemed in a hurry, so he said no more.

They were shown into a neat little sitting-room on the ground floor, but before Bertie had time to continue his inquiries, they were shown out again and requested to walk upstairs. Here they were received by a gentleman who was evidently a friend of his father's who at once began to "talk business" with him, and Bertie was left at liberty to look about him. It was a small room and rather hot, cold though the day was out of doors, for there were several people in it, writing busily or arranging papers. Bertie wondered what all this had to do with a children's hospital—it was more like father's office in Doctors' Commons, only smaller and more crowded. But he had not very long to wait—a quarter of an hour or so, and then his father called him.

"May we look at the children? It would interest my boy," he asked.

"Certainly," replied his father's friend.

Then they were asked to go upstairs again, where they were met by a sweet-faced nurse in snowy cap and apron, and Bertie began to feel as if this was the real thing at last. Sadly real—the pale-faced children in the cots were evidently much "iller," thought Bertie, than the little convalescents at the Home. They seemed comfortable and content—one or two were playing with toys and picture books, and everything was exquisitely neat and clean. But the rooms were small and dull—quite different from the high, bright "dormitories" in Bertie's memory, and there were far fewer little patients than he had expected to see. And one or two remarks he overheard added to his perplexity. "Yes," he heard the lady who was showing them over, say to his father, "the new building will not be ready a day before it is needed. It is really most distressing to be able to receive so few in winter especially there is always so much illness about here. It is heartrending to have to refuse to take in the poor little things."

"Dad," said Bertie, when they had found their way downstairs again and were standing at the front door, "Dad, I don't understand about it. It's not like a proper hospital."

"But it's going to be," said his father cheerfully, he looked up at the sky as he spoke. "It has actually stopped raining,"

he went on. "You need not open your umbrella, Bertie. Come along and I will show you what will explain it all."

They had only a few yards to go—round the corner into the Bridge Road, and there, at a little temporary door in some hoardings Bertie's father tapped for admittance. And then the boy's eyes opened wide in astonishment. A large building was in process of construction; great blocks of stone, so nice and clean, for it was new to London smoke and dirt, stood about, or old lifted to their places by gigantic cranes, workmen were busied in all directions, in spite of the gloomy weather, it was an inspiriting sight. Bertie's father showed all there was as yet to be seen.

"This will be a proper hospital, don't you think, my boy?" he asked, and Bertie eagerly answered:

"Splendid, why it's bigger than the 'Home' though, of course, there are no gardens."

"No," said his father, "that you could surely expect, though once upon a time this very spot was called "Prince's Meadows."

Bertie gazed about him. They were high up, almost as high as the roof would be, in a great unfinished space, not as yet divided into rooms and corridors. Glancing down at the endless rows of houses, it was difficult to picture the meadows of long ago. Then the boy's eyes turned to the building again.

"Don't it cost a great lot of money, dad?" he asked.

A slightly anxious look crossed his father's face.

"Ah, yes; that's the other side of the medal," he said. "An immense sum it would sound to you—thousands and thousands of pounds, and we have only got about a third of what we need."

Bertie wished to himself that some good fairy would tell him where to find a treasure cave! He told Maggie all about it when he got home, and his mother was pleased to hear how interested he was in what he had seen. But Maggie did not quite take it in at first. She cuddled up to her mother, and said she didn't see what hospitals for children were wanted for.

"I wouldn't like to be taken away to one if I was ill," she said. "I'd far, far rather stay at home and have mummy and nurse to take care of me, and I'm quite sure all children must feel like that."

"Ah, Maggie, you little know," said her mother. "Fancy being ill—very ill; your head aching so that you could scarcely lift it from the pillow—if, indeed, you had a pillow—in a small dark room, with half-a-dozen noisy brothers and sisters about, and your mother with everything to do—cooking, washing, cleaning, and the baby crying, very likely. How could a poor child get properly nursed in such a case, of which there are hundreds? Or, fancy an infant—younger than our baby, perhaps—miserably ill, and its mother obliged to go out to work, and to leave it with some little mite of a sister scarcely bigger than itself as its only nurse. Think what a blessing a children's hospital, with its large, airy rooms and clean white cots, and nice milk and soup and whatever children need when they are ill, and kind nurses and clever doctors; think what a comfort such a place must be!"

"Yes," said Maggie, "I understand now, mummy."

"But it needs such a lot of money," said Bertie, "and dad says they haven't got near enough yet."

"No," said his mother, "we are all doing what we can to collect more."

"Could we help?" asked Bertie.

"I think you might," replied his mother.

"If your schoolmasters have no objection, you might talk about it at school, and perhaps get some of your friends to collect for it in the holidays. And you, Maggie, might do something in the same way."

Both children seemed very pleased.

"I will give you collecting cards," their mother went on, "and of course you must head the lists yourselves."

"Yes," said Bertie, "I thought of that. I'm going to save all I can of my pocket-money up to Easter."

"And," added his mother, "I have thought of another thing. A little History of the Hospital has lately been written. I will give you one or two copies of it to take back to school with you, and you shall have one too, Maggie."

It remains to be seen what comes of Bertie's and Maggie's efforts. But if any of my little readers care to follow their example I can assure them that all the friends of this beautiful new hospital will be most grateful to them. And over the page you will find the little history of "Poverty Corner," with all its ups and downs.

### Croydon Infirmary and Probationers.

### THE MATRON'S SIGNATURE.

On Tuesday, at a meeting of the Croydon Board of Guardians, the Infirmary Committee reported as follows:—

Received a letter from the Clerk to the Metropolitan Asylums Board adverting to the previous correspondence on the subject of the Croydon Union Infirmary as a training school for nurses, stating that this Union Infirmary had now been added to the list of institutions recognised by that Board as training schools for nurses.

The Committee considered a petition of the probationer nurses, which was submitted to the Committee at their last meeting, and adjourned for consideration, requesting the Guardians to sanction the matron's signature to their training certificates. The Committee did not recommend a compliance with the request.

The Committee considered a letter from Mr. A. Buttle on the subject of the correspondence which had taken place between the Guardians and late Probationer Nurse Vickery, now Mrs. Buttle, on the subject of the want of the matron's signature to her certificate of training. The Committee did not see their way to re-open the correspondence in this case.

Dr. W. B. Addison moved that the recommendation as to the matron's signature be referred back to the Committee. The petition was got up entirely upon the initiative of the probationers, who took considerable trouble over the matter, and the amount of trouble taken indicated the amount of feeling.

Miss Mennell seconded the amendment.

Mr. G. J. Allen (Mayor-elect of Croydon) hoped that the Board would support the Committee on the present occasion as in the past. The absence of the matron's signature on the certificates did not do the slightest harm to the nurses, and they could not prove one case where they had been refused an appointment in consequence. He was confident that in the course of time this grievance would be forgotten. The Infirmary was now being carried on harmoniously, and it would be a great pity to attempt to go back on the resolution of the Committee.

Mr. J. W. Gardiner, J.P., said that in this matter it was the nurses who were most immediately concerned, and he did not think that they would ever let the subject drop. It would be better for the board to bring about a state of harmony, and this would not be possible until the board gave way. He suggested that those responsible for the nursing papers were responsible for this petition.

Miss Musselwhite said that the nurses had agitated about this for a long time. She believed that they had been dissatisfied with the certificates they got, and they expressed their dissatisfaction now. There was a small attendance at the meeting of the committee, and the request was only lost by the casting-vote of the chairman.

Mr. Sidey said that the point was: Was any injustice done by the present arrangement? He wanted that cleared up in his mind. Had any nurses been deprived of situations they would otherwise have got if the certificates had been signed by the matron? He also wanted to know what was the advantage of the matron signing the certificates.

Mr. Morland (Mayor of Croydon) did not think that any injustice was done. At the same time the probationers felt that the signature of the matron was something missing from the certificate which would help them get a situation. This might be a mistake on the part of the probationers. It was, however, not unnatural for the nurses to feel that, as in the main it was to matrons of institutions they applied, such matrons looked to see by what matron they were trained, and that they would demur to taking a girl whose certificate was not signed by a matron. He did not think the difficulties of administration in the Infirmary would be increased by the Board asking the matron to sign the certificates. He did not see any objection to restoring to the nurses what the latter considered an advantage, and which would probably be an advantage in some ways.

The Vice-Chairman thought from the experience of the past that the signing of certificates by a matron was placing a power in the hands of a matron which might be used to the injustice of the probationers themselves, When the petition came before the Committee not one case of injustice could be quoted. He read the letter from the Metropolitan Asylums Board recognising the Croydon Infirmary as a training school in proof that there was no injustice done. It was a mere matter of sentiment fomented from outside the Board by those conducting nursing papers.

The amendment was put and carried by 15 votes to 10. The recommendation in consequence goes back to the committee for reconsideration.

### Appointments.

[No charge is made for announcements under this head, and we are always glad to receive, and publish, appointments. But it is essential that in all cases the school of training should be given.]

Accident Hospital, Widnes.—Miss Anne Nutter has been appointed matron. She was trained at Leeds Union Infirmary, where she has since been charge nurse. She has also been charge nurse at Prescot Infirmary, staff nurse at West Ham and East London Hospital, night and also day sister at the Essex and Colchester Hospital. She holds the L O.S. certificate.

Chester Benevolent Institution.—Miss Louisa Jeaves has been appointed matron. She was trained at Plaistow, and has since been district nurse at Darwen and in East London. She has also been superintendent nurse at Chester Benevolent Institution.

Dunoon and Cowal Combination Hospital.—Miss Isobel M. Grant has been appointed matron. She was trained at Knightswood Hospital, Glasgow, and has since been nurse at the City Hospital, Edinburgh; assistant nurse at the City Hospital, Liverpool; charge nurse and assistant matron at Darnley Joint Hospital, Nitshill, Glasgow.

Eastern Counties Inebriate Home, Norfolk.—Miss Margaret Isabella Scott has been appointed assistant matron. She was trained at the Woolwich Infirmary, Plumstead, and has since been assistant superintndent at the Braintree Infirmary and charge nurse at the Dartford Infirmary. She holds the L.O.S. certificate.

East London Hospital for Children.—Miss Eleanor Inglis has been appointed sister. She was trained at St. George's Hospital, London, and the East London Hospital for Children, and has since been sister of the children's ward at Gravesend Hospital.

Ebbw Vale Isolation Hospital.—Miss Ida Maud Moss has been appointed charge nurse. She was trained at Tamworth Hospital, and has since been nurse at the City Infirmary, Birmingham, and at the Market Drayton Hospital; sister at the Brook Fever Hospital, London; nurse-matron at the Stone Isolation Hospital, Staffordshire; and charge nurse at the Isolation Hospital, Pentardawe.

Gloucester General Infirmary and Eye Institution.—Miss Kate E. Oyler has been appointed matron. She was trained at Guy's Hospital, London, and has since been sister at the National Hospital, Queen's Square, Bloomsbury, theatre and casualty sister at the Royal Portsmouth Hospital, night superintendent at the New Incorporation Infirmary, Shirley, and assistant matron at St. Mary (Islington) Infirmary. She holds the L.O.S. certificate.

High Wood School, Brentwood.—Miss E. H. Reed has been appointed charge nurse. She was trained at St. Leonard's Infirmary, Shoreditch. She has since been on the staff of the Mildmay Nursing Institution, and nurse-matron of the Invalid and Cripple Children's Home, Upper Warlingham, Surrey.

Homœopathic Hospital, Birmingham.—Miss Catherine A. Edwards has been appointed night charge nurse. She was trained at the Hahnemann Hospital, Liverpool, where she took temporary night sister's duty, and has since been Queen's nurse at Ashton-under-Lyne,

Horwich, Westhoughton, and Blackrod Hospital, Fall Birch, Horwich.—Miss Sarah Edith Hughes has been appointed matron. She received general training at Tonbridge Infirmary and fever training at Brighton Hospital. She has since been charge nurse at Tonbridge Infirmary, superintendent nurse at Battle Union, matron in charge of Hawarden Isolation Hospital, and assistant matron at Parkhurst, Newport, Isle of Wight. She has also done private nursing.

Iver Langley and Denham Cottage Hospital.—Miss Marion Savell has been appointed nurse-matron. She was trained at University Cottage Hospital, London.

Manchester Jewish Hospital.—Miss Annie E. Ivin has been appointed staff nurse. She was trained at the Guest Hospital, Dudley. She has since been head nurse at Lipton Urban District Hospital, and has done private nursing.

National Hospital for Consumption, Ventnor.—Miss Christina Cameron has been appointed matron. She was trained at the Royal Infirmary, Dumfries, and has since been assistant matron at the Victoria Park Chest Hospital, London.

Parish of St. John, Hampstead Workhouse Sick Wards.—Miss Matilda Florence Miller has been appointed charge nurse. She was trained at Camberwell Infirmary and has since been nurse at the South Western Fever Hospital, London.

Queen Victoria Nurses' Home, Sheffield.—Miss Mary G. Milne has been appointed superintendent. She was trained at Hull Royal Infirmary and for district work under the Cardiff branch of the Queen Victoria Jubilee Institute. She has since been Queen's nurse at Gateshead and Bingley, and for the last six months she has been taking temporary duty as superintendent nurse of the Queen's Home at Darwen.

Royal Hospital for Incurables, Donnybrook, Dublin.—Miss L. A. Brabazon has been appointed night superintendent. She was trained at Sir Patrick Dun's Hospital, Dublin, and has since been staff nurse at the Royal Victoria Hospital, Belfast.

Royal Victoria Hospital, Belfast.—Miss Kathleen Haire has been appointed staff nurse. She was trained at Sir Patrick Dun's Hospital, Dublin, worked for some time on the private nursing staff, and was temporary ward and night sister.

Spittlesea Hospital, Luton.—Miss Sadie Hackney has been appointed nurse-matron. She was trained at the Royal Hospital, Belfast, and since been supernumerary and charge nurse at Edinburgh Royal Infirmary, charge nurse at Park Hospital, Hither Green; sister at the City Fever Hospital, Ruchill, Glasgow; and charge nurse at the North-Eastern Hospital, London. She has also done private nursing, matron's duty at the Convalescent Home, Gilmerton, and has had entire charge of the Boys' Sanatorium, Uffculme, Devon, during an epidemic of scarlet fever.

### TRAVEL NOTES AND QUERIES.

By our Travel Correspondent.

Passage to Canada (Canada).—By the Allan Line the cost of a first-class ticket to Quebec and Montreal is £11; second class, £7 10s. Write to Messrs. Cook, Ludgate Circus, and ask them for all information as to the cheapest way of reaching Assimiboia and whether there is any possibility of getting an assisted passage. I cannot answer that question myself with decision, but as you are meaning to take up work I think most likely you will be able to benefit by such assistance.

Winter in the South (Lonsdale).—A villa, however small, either on the Riviera or in Pau would be expensive. I should think the cost would be nearly double that of living in a reasonable hotel or pension. It is not merely the housekeeping, in that probably you would find little difficulty, though marketing abroad is an art in itself and a very different matter to catering for a large public institution. First of all the rents are immensely high where you wish to go, because owners must make a harvest in their short season; then there are several small taxes and unexpected expenses too numerous to mention. Then the cost of replacing injured and broken items in the inventory—these trifles have a marvellous way of accumulating; then your *femme à faire tout* is to be reckoned with, and, finally, as a householder you will be asked to contribute to many things which as a lodger you would escape. If you can afford what you say, you can live most comfortably on the Riviera or in Pau. There is much more variety at the former place. I should suggest Mentone or San Remo; though Italian towns, French will be equally useful to you. Pau is a shorter journey, but there is not so much variety. San Jean-de-Luz is a nice little place, but much quieter than the Riviera. Tell me which of these four places you like best and I will give you addresses.

Rules in Regard to Correspondence for this Section.—All questioners must use a pseudonym for publication, but the communication must also bear the writer's own name and address as well, which will be regarded as confidential. All such communications to be addressed "Travel Correspondent, 'Nursing Section of *The Hospital*,' 28 Southampton Street, Strand." No charge will be made for inserting and answering questions in the inquiry column, and all will be answered in rotation as space permits. If an answer by letter is required, a stamped and addressed envelope must be enclosed, together with 2s. 6d., which fee will be devoted to the objects of "The Hospital" Convalescent Fund. Ten days must be allowed before an answer can be published.

### Everybody's Opinion.

[Correspondence on all subjects is invited, but we cannot in any way be responsible for the opinions expressed by our correspondents. No communication can be entertained if the name and address of the correspondent are not given as a guarantee of good faith, but not necessarily for publication. All correspondents should write on one side of the paper only.]

### INSPECTORSHIPS.

Sir William Chance says:—I am glad to see that your remark as to the inspection of boarded-out children did not refer to the inspectors of the Local Government Board. Doubtless owing to my bad writing you transform Miss Mason, the senior inspector of boarding-out, into Miss Ward, the Lewis inspector of boarding-out, who is, I fancy, non-existent. I hope you will insert this correction in your next issue.

### THE PRESENTATION CRAZE.

"G. B." writes: May I be allowed through the medium of your valuable paper to call attention to an abuse which seems to be getting more prevalent every year in hospitals and other similar institutions. I refer to the presentation or testimonial craze, which threatens to become a serious drain on the too often scanty income of the nurse. It would be astonishing, if they were totalled, how many times in a year the slip of paper comes round to nurses for contributions for some individual in the hospital who is leaving. At Christmas-time especially there are many recipients of the nurses' bounty, perhaps a present for the matron and the sister of the ward, the wardmaid, porters, etc. I recollect a collection being taken from the nurses of a certain hospital to get a house-porter—who was not leaving the institution—a wedding present. Another flagrant instance of this odious custom was when a resident doctor was promoted to an appointment on the outside staff of the hospital, a presentation was mooted and carried out. I think that matrons, sisters, and others in authority should discourage the practice of giving and receiving presents at Christmas and at other times, unless the circumstances are very exceptional. I have reason to believe that subscriptions for presentations are often grudged, and nurses who cannot afford it give because otherwise they might be snubbed by their richer confrères.

### THE STANDARD OF PRIVATE NURSING.

"M. E. B." writes: I should like to say that I entirely agree with "An Ordinary Nurse." Private nursing in my opinion calls for the best that is in us. In many ways it is far more trying than hospital work. There is less physical, but much greater mental strain. Then, amongst a large number of patients it is obvious that a few minutes only can be spent at the bedside of each one, whereas the greater part of the 24 hours must be spent with one patient in a private house, the patient must be kept cheerful and all their little fads remembered. Have we not all, in our time, been somewhat wearied by the "little fads" of our private patient? Private nursing calls into play three essentially womanly qualities, tact, sympathy, and, above all, patience. The private nurse has countless difficulties to contend with, and if she can overcome them and still preserve a cheerful spirit, then I maintain that she is a woman to be admired. In private nursing we know that it is the nurse alone on whom the patient depends to help him through the pain and daily trials of a long illness, and the almost as trying period of convalescence. It is a nurse's privilege to do this to the best of her ability, not, as I read to my sorrow in a former article, because she is the "paid servant" of the patient, but because she is a woman—and this is woman's work.

### VACANT APPOINTMENTS AND ORIGINAL TESTIMONIALS.

"Advertiser" writes:—I advertised in The Hospital for a nurse. Although the case was not made attractive and the salary mentioned was not higher than would be usual, I

received nearly 200 replies. The first few received and opened afforded ample material to follow up, but as I found that some of these contained stamped envelopes and even original testimonials and photographs, I felt compelled to go through the whole lot in order to return these valuables. One good lady had even enclosed five penny stamps, with what object she did not say. Will you allow me to indicate that I am just as anxious to obtain a suitable nurse as the applicants seem to be to secure a suitable position; but that the fact of there being enclosed a stamped envelope does not make me more ready to correspond, if the essentials of ample particulars and qualifications are wanting. One is compelled, however, to send back the stamped envelope however unsuitable the applicant may be; and in the case of original testimonials and photos being forwarded, it only seems charitable to return these, even when the stamped envelope is omitted. It would be expensive, indeed, if the precedent were established, and I should be deterred from using advertising to supply my wants hereafter.

[Our correspondent's letter points a moral. Neither original testimonials nor photographs should be sent by applicants for an appointment, except by special request, and stamped envelopes are always unnecessary. — Ed. Hospital.]

### CLASS LEGISLATION.

"One who Cannot be Registered" writes: In reply to "Inquirer's" letter may I say that I do not know where she has seen the statement that nurses who have been trained at infirmaries or in small hospitals will not be eligible for registration. As I understand it, when registration is an accomplished fact any nurse will be eligible for State registration who can show that she has spent the necessary time in hospital or infirmary wards, and whose training school certifies her to be suitable as regards character. It is quite true that many of the smaller institutions will find to their cost that their nurses are unable to pass the State examination owing to the inadequate training they have given them. But these institutions will then be forced to evolve some scheme of co-operation with larger infirmaries and hospitals by which their nurses will be able to be passed on from one to the other. This will be very much to the advantage of the probationer, who, as matters now stand, often finds out too late that the institution to which she has bound herself can by no means give her an all-round nursing education. "Inquirer" expresses surprise that so many nurses trained at small places are agitating for State registration, taking for granted as she does that these will be excluded from the roll. If State registration becomes an accomplished fact, as I sincerely trust it will, we may feel certain that, as has been done in the Midwives Act and as has been done in the Registration Act in New Zealand, a term of grace will be granted, and we nurses, whose only fault is that we have been born a little too early in the century, will find that an open door has been provided for us, and that we shall not suffer. Even if it were not so, there are many of us who have the honour and the welfare of our profession enough at heart to be willing to sacrifice something to see it put on a better footing.

### NURSES FOR THE MIDDLE CLASSES.

"H. M." writes: I have read with much interest your article on "Nurses for the Middle Classes." Such a scheme of Visiting Nursing has been tried by my partner and myself during the past 16 months, and our experience may prove of some interest to your readers. We have a surgical and medical Home in a good locality in the South-West of London, and it occurred to us that it would be an excellent plan to have a thoroughly reliable nurse (not too young) on our salaried staff, to pay daily visits to people ill in flats or apartments, when a resident nurse was only an incubus. With this object we approached the doctors who attend the Home, and one and all agreed that it was one of the crying needs of this age of "Flatdom." Accordingly we sent out circulars and terms to the whole neighbourhood, stating what we were prepared to undertake. We are surrounded by flats and small houses, which are let out in apartments; to all of these we sent notices, also to every house in the large streets and squares. The results have been noteworthy! Out of the sowing of some thousand circulars, we have reaped six cases. Four of these cases were sent us by doctors. One, a lady in a hotel with a maid-attendant; another, a young shop assistant very ill in one small room, but not the class for a general ward; another, a lady in rooms who suffered from periodical bad headaches, and required a nurse for a whole day when ill; the fourth, a woman who required a visit once a week. The remaining two out of the six cases came from our circulars, and curiously enough in each case the call was from a large house in a fashionable square (one, an old paralytic lady who required attendance for two hours daily, while her maid-attendant went out; the other, a lady who was to have a consultation in her own house, and required a nurse to attend for that one morning and get everything ready). We still retain our nurse and are still at all times ready and willing to alleviate pain and meet the requirements of those unable to engage, be the reason what it may, a resident nurse. Our doctors still inform us that there is the crying need for such work in this particular neighbourhood. It may be true, but certainly the facts I have laid before you go to prove either that the contrary is the case or that the English public are wondrous slow to grasp a new method, which if supported, would be of permanent benefit to themselves. The same applies to the cubicle ward in this Home, which we started a year ago for gentlewomen of limited means. Our charge was three or four guineas, and for this everything was provided. We were assured that it would be a boon and meet the question of "hospital abuse." This ward has been widely circulated, but though our home has been full in a year we have had only one case for the cubicle ward. Now it is withdrawn, and we have utilised the accommodation for private rooms. In your article you say there should be "homes" from which nurses can work, also that they "should be subjected to inspection." We are perfectly willing to be one of the said homes, and more than willing for the inspection; in fact, speaking as the superintendent of a nursing home, I think that every nursing home should be registered and inspected by Government. Such a course would be the means of eradicating those homes which are unfit to be so named, and be fairer to those who loyally and honourably combine the task of tending the sick with earning a livelihood.

### Novelties for Nurses.

By Our Shopping Correspondent.

### WARM CLOTHING.

It would be difficult to speak in higher terms than deserved of the textures manufactured by Messrs. Greensmith, Downes and Son, of George Street, Edinburgh, and known as "Australama," "Llanola," and "Macgregor's Scotch Wincey." "Australama" is a mixture of finest Australian wool and Indian cashmere. It is made in a variety of ways to suit all requirements, from the finest underclothing or for the thickest and warmest of under and upper garments. It is soft, elastic, porous, and non-shrinking. The garments to be procured at the manufacturers are exceptionally well-made and finished. The ladies' knickerbockers are quite the nicest I have seen, and their durability is beyond doubt. They should convince the wearers of that antiquated garment, the flannel-petticoat, of the advantages of its bifurcated successor, which permits of a freedom of action combined with warmth, which cannot otherwise be experienced. "Llanola" is a material very similar to Australama, but it does not contain Indian cashmere, it is in consequence not quite so fine. Garments of either material will be found economical because both are thoroughly good, and will outwear numbers of cheap and badly-made rivals. The Macgregor wincey is an ideal material for warm nightgowns. It is very fine and close in texture, and the surface is smooth and less irritating than most woollen materials. The fancy winceys are very pretty, and suitable for blouses and costumes.

### Echoes from the Outside World.

### Movements of Royalty.

On Wednesday the King celebrated his 63rd birthday at Sandringham. There were general manifestations of enthusiasm in the country. The large body of workpeople upon the royal estate were entertained at dinner in a marquee, which was erected in the park. All the food provided for the guests was cooked as usual at Sandringham House. The Queen, the Prince and Princess of Wales and their children, Princess Victoria, and Prince George of Greece are at Sandringham. Last week his Majesty visited the Dersingham schools, where he displayed much interest in examining scholars in their reading and writing exercises, and also looked over several of their books. The visit was quite a surprise to teachers and scholars alike. He also inspected the new playground for the school children, which had been laid out at his Majesty's expense, and expressed his satisfaction at the way in which his desires have been carried into effect.

### Birthday Honours.

Among the appointments made by the King on the occasion of his Majesty's birthday are the bestowal of a baronetcy upon Mr. Michael B. Nairn, of Kirkcaldy, a generous contributor to charitable objects, and a founder of the cottage hospital and technical schools; and of knighthood; upon Mr. R. M. Beachcroft, chairman of the Metropolitan Water Board; Mr. Astor Webb, R.A., the designer of the general scheme of the Victoria Memorial in the Mall and in front of Buckingham Palace; Dr. Charles Marriott, consulting surgeon at Leicester Infirmary, and founder of the nursing institution; Dr. Shirley Murphy, medical officer to the London County Council; Professor Sinclair, professor of obstetrics and gynæcology at Manchester University; and Dr. Swan, the inventor of the Swan electric lamp.

### The Presidency of the United States.

Mr. Theodore Roosevelt has for the second time been elected President of the United States. His opponent, the Democratic candidate, was Judge Parker, but the majority cast for Mr. Roosevelt, the Republican candidate, was enormous. The public did not vote directly for President, but for the Presidential electors, who make the final election. This act of the "Electoral College," as it is called, is, however, a mere formality. Sixteen million voters had the opportunity of polling between sunrise and seven o'clock.

### The Anglo-Russian Agreement.

It is announced that the Russian Government has accepted the draft of the Convention proposed by Great Britain regarding the Commission to investigate the North Sea incident. The Commission is to report on all the circumstances relating to the disaster, and to establish the responsibility. It is to consist of five members, four of them officers of Great Britain, Russia, the United States, and France respectively. The fifth Commissioner is to be appointed by agreement between them, and if they cannot agree the choice is to be entrusted to the king of a country subsequently to be determined upon. The Commission is to meet in Paris.

### The War in the Far East.

The birthday of the Mikado was celebrated last Thursday at Tokio and throughout Japan with great enthusiasm. His Majesty held a review of troops, and afterwards gave a luncheon to the diplomatic body and the high State officials. Welcoming his guests, he expressed regret that the time had not yet come to see peace in the Far East restored in realisation of his desire. The Belgian Minister, in his reply, reciprocated the Mikado's desire for peace.—Generals Kuropatkin and Sakharoff report skirmishes at various points of the front on the Sha-ho.—An official telegram from Tokio supplies an account by the commander of the army before Port Arthur of the fighting from October 30th to November 3rd. The Japanese have captured several forts, and have brought heavy guns to bear on the harbour and shipping, sinking several steamers and causing conflagrations in the neighbourhood of the docks. The Tsar is said to have placed £4,000,000 at the disposal of the Russian Admiralty from his private purse.

### Conference of Women Workers.

The National Union of Women Workers is holding its annual conference at York this week. The preliminary meeting took place on Monday, and the subject of girls' clubs, and the best methods for the workers to employ came under discussion. The President spoke of the desirability of the educated girl sharing the good things of her life with the working girl. A delegate from London alluded to the fact that the dread of the future held by the working girl, unless she married, was largely responsible for the absurdly early marriages which all deplored, and hoped that the club by giving a higher standard, might act as a deterrent; whilst another speaker advocated military drill, and was in favour of giving the lads and elder girls opportunities to meet in public under wise supervision. Mrs. Maclagan, wife of the Archbishop of York, welcomed the Conference to the city on Tuesday, and Mrs. Clifford, of Bristol, then delivered her presidential address, in the course of which she pleaded for consolidation among women workers all over the world, in order to get their aims and objects more definitely to work. The whole day was devoted to the discussion of hygiene in its various phases.

### The Sale of the "Standard."

It was announced on Saturday that the *Standard* had been sold to Mr. C. Arthur Pearson. The great Conservative newspaper was started in the year 1829, and was first published as an evening issue in order to oppose Roman Catholic emancipation. Subsequently the *Evening Standard* became subsidiary to the morning journal. The original price was 4d., and its first editor was Dr. Giffard, a very accomplished man, under whose auspices it made rapid progress. Mr. Frank Baldwin, son of the founder, sold it for £16,500 to Mr. James Johnston, who reduced the price to 2d., and in 1858 to 1d. It remained in the hands of Mr. Johnston's family after his death until now. Mr. Pearson intimates that no alterations are contemplated in the price, appearance, or general tone of the paper.

### The Antarctic Exhibition.

At the Bruton Galleries, Bruton Street, Bond Street, an interesting little collection is now on view illustrating the sojourn of the *Discovery* in the South Polar seas. Engineer-Lieutenant Skelton was the chief photographer of the party, and the 175 items which he contributes portray vividly their movements and experiences. There are numerous views of Antarctic scenery, icebergs, glaciers, the return of sledge parties, a bird's-eye view of a seal rookery, and "King Penguin's Rookery," showing crowds of the great solemn, white-robed birds going across the ice near Cape Crozier in stately procession. Dr. Edward Wilson has on show a number of water-colour landscapes, landscapes in which ice of every hue and shade is the principal feature. Some of the colour effects are marvellous. In addition to the pictures, there is a model of the good ship *Discovery* herself, as well as samples of the gear the explorers carried, the clothes they wore, the rations they ate, the bags they slept in, as well as their sledges, snow-shoes, snow-goggles, and even the teeth of the last Eskimo dog which died on Captain Scott's final journey.

### Notes and Queries.

**FOR REGULATIONS SEE PAGE 12.**

*The Naval Nursing Service.*

(43) Will you kindly tell me how I could get into the Royal Naval Nursing Service?—*Jean.*

Write to the Director-General, Medical Department of Navy, Admiralty. Also see "The Nursing Profession: How and Where to Train."

Will you kindly tell me the address for obtaining rules and particulars of Navy nursing?—*Wee McGregor.*

See reply to *Jean.*

*Army Nursing Reserve.*

(44) I should be glad to know whether there is any chance of a Reserve Sister's pay being placed on an equal footing with that of the Sisters of the Queen Alexandra's Imperial Military Nursing Service? Many of the Reservists are now working under the new regulations, taking the combined duties of sisters and staff nurses, although their pay does not equal that of a regular staff nurse, their services may be dispensed with at any time, and they get no pension.—*A. N. S. R.*

Decidedly there is "a chance" of a Reserve Sister's pay being placed on an equal footing with that in corresponding ranks of the Regular Service, but as yet no definite statement has been made by the War Office. You are not correct in stating that "the pay of a Reserve Sister taking the combined duties of staff nurse and sister does not equal that of a regular staff nurse." A nurse in this position receives the same rate of pay as a staff nurse entering the Service *plus* £20 abroad and £10 at home gratuity for every year of service. A staff nurse in Queen Alexandra's Imperial Military Nursing Service has no gratuity if she leaves, and no pension until she has attained the age limit.

*Lady Dufferin's Fund.*

(45) Will you kindly tell me to whom I must apply to obtain full particulars of Lady Dufferin's training for nurses for service in India?—*E. C.*

Write to Miss Manning, of 5 Pembridge Crescent, Notting Hill Gate, W., for any particulars of the National Association for Supplying Female Medical Aid to the Women of India, which was founded by Lady Dufferin.

*Books.*

(46) Can you kindly give me any information as to the most suitable books to use in preparing for the assistant's examination the Society of Apothecaries, London?—*E. L. B.*

Write to the Secretary, the Pharmaceutical Society, 17 Bloomsbury Square, W.C.

*Male Nurse.*

(47) (1) I should feel obliged if you could inform me of any asylums which employ male companions. I am 24 years of age and have had two years' training in a large asylum. I have already applied to the medical superintendent of N——, but there is no vacancy. (2) Can you supply me with the names of hospitals in London for the training of male nurses, and give me particulars as to length of training, salary, etc.?—*Male Nurse.*

(1) You could only obtain this information by advertising. (2) The National Hospital for the Paralysed and Epileptic, Queen's Square, Bloomsbury, W.C., is the only civil hospital for training male nurses. The course is for two years; the salary is £12 for the first and £20 for the second year.

*Sanitary Inspector.*

(48) Can you direct me where to obtain information as to the post of lady sanitary inspector? After securing the certificate is there any difficulty in obtaining a post? Is the profession overcrowded, and how are the appointments made?—*Laura.*

Write to the Secr tary, the Sanitary Institute, Margaret Street, W. The demand for lady sanitary inspectors is increasing, but competition is very keen whenever a post is advertised.

**Handbooks for Nurses.**

"The Nurses' Pronouncing Dictionary." 2s. post free.

"Surgical Ward Work and Nursing." By Dr. A. Miles. Price 3s. 6d. net; 3s. 10d. post free.

"The Human Body: Practical Physiology and Hygiene." 5s. post free.

"The Nursing Profession: How and Where to Train." 2s. net; 2s. 4d. post free.

"Preparation for Operation in Private Houses." Price 6d. post free.

The above may be obtained of all booksellers or of The Scientific Press, Limited, 28 & 29 Southampton Street, Strand, London, W.C.

### For Reading to the Sick.

LOOKING UNTO JESUS.

LORD, as to Thy dear cross we flee
And plead to be forgiven;
So let Thy life our pattern be,
And form our souls for heaven.

Help us through good report and ill,
Our daily cross to bear;
Like Thee to do our Father's will,
Our brother's grief to share.

Let grace our selfishness expel,
Our earthliness refine,
And kindness in our bosoms dwell,
As free and true as Thine.

Kept peaceful in the midst of strife,
Forgiving and forgiven,
O may we lead the pilgrim life
And follow Thee to Heaven.

*John Hampden Gurney.*

God has given each one of us a life to live, a burden to bear, a problem to work out. How are we working out the problem, how are we bearing the burden?

Are you then looking up to Him for His approval of your daily life and work? Are you endeavouring to bring your whole life under the discipline and directing force of His inspiration?

If so, all is well, for He will not be overmuch cast down by apparent failure, by misunderstanding, by loss of apparent influence, by reverses, by d saster, by catastrophes.

You will remember that what He always and for ever asks for, is faithful performance of that which He gives us to do. Whether it be to bear the burden or to live. If the Cross says anything, it says that apparent defeat is often real victory, that true and noble failures are proclaimed to be triumphs in the "It is finished" of Jesus Christ.

*Randolph.*

Every struggle wrought out in humble dependence on the Lord Jesus Christ shall be reckoned up at the last day as something done unto Himself.

And when we look around, and are bewildered at the apparently unequal distribution of suffering among God's servants, how some serve Him in peace and gladness through their long untroubled summer day, and some are pierced through and through with bitter sorrows, it is surely no small compensation to the burdened ones to feel that they may be fulfilling for some weaker members the suffering under which these would have sunk. "Gather my saints together unto Me, saith the Lord; those that have made a covenant with Me by sacrifice."—*Mrs. C. Campbell.*

O Lord, we pray Thee that the thought of the country towards which we are travelling may make us forgetful of the weariness of the journey; and if Thou addest a weight of troubles, give us also strength that we may not faint under the burden. Thou, O Lord, hast gone before us, bearing Thy Cross, make us to bear ours after Thee, ever looking unto Thee, and putting our trust in Thee, O Lord alone.—*Anon.*

